# Follow‐Up Effect of Wechat Group on Discharged Patients with Colles Fracture

**DOI:** 10.1002/gch2.202300070

**Published:** 2023-05-16

**Authors:** Lifen Tang, Jingjing Dai

**Affiliations:** ^1^ Department of Orthopaedics Shanghai Ninth People's Hospital Shanghai JiaoTong University School of Medicine 639 Zhizaoju Road Shanghai 200011 P. R. China; ^2^ Nursing department Shanghai Ninth People's Hospital Shanghai JiaoTong University School of Medicine 639 Zhizaoju Road Shanghai 200011 P. R. China

**Keywords:** Colles fracture, continuing care, follow‐up, Wechat groups

## Abstract

This work explores the effect of a Wechat group on follow‐up and continuation of nursing for discharged patients with Colles fracture. A total of 96 patients with Colles fracture are enrolled and randomized into two groups by the random number table method. The control group is followed up and guided by traditional methods. Based on the follow‐up method adopted in the control group, a Wechat group is combined with Wechat constant tracking. The regular follow‐up rate, a satisfaction of with continuing care, Gartland–Werley wrist score, the exercise of self‐care agency score (ESCA score), and complications are compared and analyzed. The regular follow‐up rate, satisfaction, Gartland–Werley wrist score, and self‐care ability of patients in the Wechat group are significantly higher than those in the control group (*p* < 0.05). Statistical analysis of postoperative complications showed that although there is no significant difference in the incidence of median nerve irritation and incision infection (*p* > 0.05), the proportion of joint stiffness in the control group is significantly higher (*p* < 0.05). The establishment of Wechat groups to follow up and continue nursing for discharged patients with Colles fracture is helpful to achieve better clinical efficacy and improve the effective way for doctor–patient communication, which is worthy of active promotion.

## Graphical abstract

This work explores the effect of a Wechat group on follow‐up and continuation of nursing for discharged patients with Colles fracture. The regular follow‐up rate, satisfaction, Gartland–Werley wrist score, and self‐care ability of patients in the Wechat group are significantly higher than those in the control group.

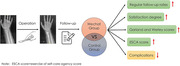

## 1 Introduction

Fractures of the distal radius account for 16% of all fractures and are the most prevalent injury that orthopaedic surgeons have to manage,^[^ ^1^ ^]^ with Colles fractures being the most common type.^[^ ^2^, ^3^ ^]^ It mainly occurs in middle‐aged and elderly patients, and females are more than males. The most common cause of Colles fracture is a fall in which the elbow of the affected limb is extended, the forearm is pronated, the wrist is extended back, and the palm is on the ground. The severity of the injury is usually determined by the position of the wrist at the time of injury and the force of the trauma. The influence of trauma through the wrist produces bending and compression forces, resulting in a fracture at the weak area of the distal radius, which is characterized by displacement of the distal end to the radial dorsal and proximal end to the volar side. Most Colles fractures can be treated by manual reduction and plaster fixation unless the fractures comminuted with apparent displacement, involved the articular surface of the lower radius, or challenging to keep the manual reduction, in which case surgical treatment is necessary.^[^ ^4^, ^5^ ^]^ Because this kind of operation is relatively simple and the risk is small, most patients will be discharged from hospital within a day or two days after the procedure, resulting in the postoperative recovery and functional exercise of these patients can only be carried out outside the hospital by themselves. Therefore, the prognosis of different patients often varies greatly due to lack of continuous and professional guidance.

Wechat is a free application that provides instant messaging services for intelligent terminals. It supports cross‐communication operators and cross‐operating system platforms, and uses the web to send free voice messages, videos, images, and comments quickly as well as call function. It has the advantages of low cost, flexibility, and simplicity.^[^ ^6^ ^]^ Wechat public account provides an interactive platform between organizations and users.^[^ ^7^ ^]^ Therefore, to allow patients to get regular guidance and professional supervision, we established a Wechat group of discharged patients with Colles fractures for regular follow‐up and nursing rehabilitation guidance. Through the compliance of patients with functional exercise, functional recovery degree, and nursing work satisfaction, we further explored the application value of Wechat group combined health guidance in the prognosis of Colles fracture. The correlation analysis is as follows.

## 2 Results

### 2.1 Comparison of Regular Follow‐Up Rates between the Two Groups

The time points of follow‐up were 1 month, 3 months and 6 months after surgery. The completion of three follow‐up visits was defined as regular follow‐up, otherwise it was considered irregular follow‐up. The number of patients who did not regularly follow up was 1 in the Wechat group and 12 in the control group. Namely, the regular follow‐up rate was 97.9% in the Wechat group and 75.0% in the control group. The difference was statistically significant (*p* < 0.05), as shown in **Table** **1**.

**Table 1:** Comparison of regular follow‐up rates. The number of patients who regularly follow up was 47 in the Wechat group and 36 in the control group. The Chi‐square test was adopted, revealing that the difference was statistically significant (*p* < 0.05)

**Group**	**Regular Follow‐Up**	**Irregular follow‐up**	***χ*2**	***P***
Control group	36	12	10.766	0.001
Wechat group	47	1		

### 2.2 Comparison of Patients' Satisfaction with Continuing Nursing Work between Two Groups

The satisfaction questionnaire was used to record patients' satisfaction in the two groups with continuing care after discharge. And patients with regular follow‐up were selected for analysis. The sample size was 47 in the WeChat group and 36 in the control group. Each patient will be queried 3 times. And satisfation should get all satisfatory feedbacks, while average should get one or more average feedback except statifactory feeback. Any dissatifactory feedback is given will be classified as dissatisfaction. Statistics showed that the satisfaction of patients in the Wechat group was significantly higher than that in the control group, with statistical significance (*p* < 0.05), as shown in **Table** **2**.

**Table 2:** Comparison of satisfaction with continuing care. The sample size was 83, of which the satisfaction, average and dissatisfaction case data in the WeChat group and control group were expressed in total number of cases. Wilcoxon rank sum test was used. The significance test level was *p* < 0.05 as the difference was statistically significant

**Group**	**Satisfaction**	**Average**	**Dissatisfaction**	***Z***	***P***
Control group	10	23	3	−2.656	0.008
Wechat group	28	16	3		

### 2.3 Comparison of Garland and Werley Scores between the Two Groups

Gartland–Werley wrist score was used to evaluate the function of the two groups. The score included the degree of residual radial deviation and dorsal tilt deformity, subjective evaluation of pain, mobility limitation or loss of function by patients, objective evaluation of wrist flexion, extension, and rotation and grip strength by physicians, and the proportion of complications such as arthritis, median nerve injury and finger dysfunction. According to the evaluation system, wrist function was divided into excellent (0–2 points), good (3–8 points), fair (9–20 points), and poor (≥21 points). The excellent and good rate (EGR) of the Wechat group was 83.0%, which was significantly better than that of the control group (63.9%), and the difference was statistically significant (*p* < 0.05), as shown in **Table** **3**.

**Table 3:** Comparison of Garland and Werley wrist scores. Patients with regular follow‐up were selected for analysis. The sample size was 83. The number of patients who achieved excellent and good recovery of joint function 39 in the Wechat group and 23 in the control group. The excellent and good rate was 83.0% in the Wechat group and 63.9% in the control group. The Wilcoxon rank sum test was used, showing a statistically significant difference between the two groups

**Group**	***n***	**Excellent**	**Good**	**Fair**	**Poor**	**EGR**	***Z***	***P***
Control group	36	13	10	9	4	63.9%	−2.082	0.037
Wechat group	47	25	14	8	0	83.0%		

### 2.4 Comparison of the Exercise of Self‐Care Agency Score (ESCA Score) between the Two Groups at 3 Months after the Operation

The patients were followed up 3 months after the operation, and the exercise of self‐care agency scale was given to the two groups to fill in, and then statistical analysis was performed. The results showed that the ESCA score of the Wechat group was higher than that of the control group, and the difference was statistically significant (*p* < 0.05), as shown in **Table** **4**.

**Table 4:** Comparison of ESCA score. 83 patients with regular follow‐up were selected for analysis. The t‐test was adopted with the results showing that patients in the Wechat group were significantly better than the control group in terms of self‐care ability (*p* < 0.05)

**Group**	**x ± s**	***t***	***P***
Control group	119.50 ± 6.281	−13.664	<0.001
Wechat group	135.40 group		

### 2.5 Comparison of Complications between the Two Groups

All patients were followed up to 6 months after surgery to observe the occurrence of complications. And then the postoperative complications of the two groups were recorded. It was found that median nerve irritation and incision infection had no significant statistical difference between the two groups (*p* < 0.05). In contrast, the incidence of joint stiffness in the Wechat group was significantly lower than that in the control group (*p* < 0.05), as shown in **Table** **5**.

**Table 5:** Comparison of complications. 83 patients with regular follow‐up were selected for analysis. The Chi‐square test was used. Statistical analysis showed no statistical difference in the incidence of median nerve irritation and incision infection, but joint stiffness in the control group was significantly higher than that in the Wechat group (*p* < 0.05)

**Category of Complications**		**Control Group**	**Wechat Group**	***χ*2**	***P***
Median nerve irritation	Yes	4	2	0.589	0.443
	No	32	45		
Incision infection	Yes	3	2	0.095	0.758
	No	33	45		
Joint stiffness	Yes	10	3	7.064	0.008
	No	26	44		

## 3 Discussion

Fractures of the distal radius are the most common type of fracture worldwide and tend to occur in older patients but also patients of all ages.^[^ ^8^ ^]^ Colles fracture is the most common fracture of the distal radius. Although conservative treatment is satisfactory for most patients, surgery is necessary to solve the severe fracture type. The common surgical intervention method in clinical practice is open reduction and internal fixation with bone plate and screw, which can achieve a good fracture reduction effect.^[^ ^9^ ^]^ However, due to a lack of convenience and timely contact means in the past and relatively lower risk and severity of Colles fracture, many patients do not pay much attention to the disease and follow up on time, resulting in the inability to get regular rehabilitation exercise guidance. Even if the operation is very successful, some patients fail to achieve good functional recovery. Although with the increasing awareness of health nowadays, people are willing to spend more time on rehabilitation exercises. However, due to the lack of necessary basic medical knowledge, the traditional telephone or outpatient follow‐up cannot solve all the needs of patients. The same problem may need to be explained repeatedly, which wastes the human resources, material resources, and time of doctors and nurses. To solve such problems, our team introduced Wechat platform, a convenient communication medium, into the continuous care and rehabilitation exercise after Colles fracture surgery, which is also a new attempt to explore the constant follow‐up mode in the information age.

Our results show that communication and reminders through Wechat groups can significantly improve patients' regular follow‐up rate compared with traditional follow‐up methods. The end of treatment does not end with the patient being discharged from hospital, but with a continuation of a new pattern. Patients can get the timely guidance from the medical and nursing team, which can improve patients' satisfaction with the treatment effect, promote the harmony of the doctor‐patient relationship, and finally make patients gain greater benefits in the recovery process. Specifically, through the guidance of Wechat group, patients' functional exercise after discharge is timelier and more regular. According to the evaluation of the Garland and Werley scoring system, compared with the control group, patients in the Wechat group had a higher excellent and reasonable rate of postoperative function, better self‐care ability, and fewer complications that had significant statistical differences.

In conclusion, the application of Wechat group combined with health guidance nursing intervention in patients with Colles fracture can effectively improve patients' disease awareness, functional exercise compliance, and self‐care ability, improve nursing job satisfaction, and help to regulate patients' psychological state, which is worthy of clinical promotion.

However, the current study is just a single‐center study. Further analysis of the effect may need to include more centers and more patients. In addition, the follow‐up mean involved in this study can only help patients who have the ability to use wechat, but cannot benefit all patients, which is an important content for us to think about and improve in the future.

## 4 Experimental Section

### The General Information

Ninety‐six patients with Colles fractures treated in the hospital from January 2020 to August 2022 were selected as the research participants. According to the random number table method, they were divided into Wechat group and a control group, with 48 cases in each group. Inclusion criteria were as follows: over 18 years of age, male or female, clinical diagnosis consistent with colles fracture, able to use wechat, and voluntary participation in the study. There were 7 male and 41 female patients in the Wechat group, aged 52–77 years. In the control group, 11 male and 37 female patients aged 48–69 years. There was no significant difference in gender, age, cause of injury, and severity of illness between the two groups (*p* > 0.05). Therefore, the two groups were comparable. All participants signed informed consent and volunteered to participate in the program, which the ethics committee approved (approval number: SH9H‐2021‐T182‐1).

### Follow‐Up Strategy

*Wechat Group*: Based on the follow‐up method adopted in the control group, Wechat group was combined with constant follow‐up, and health guidance intervention was given. Specific methods are as follows:

1) A dedicated Wechat account was applied for follow‐up and guidance. To protect the privacy of patients, a separate Wechat group of rehabilitation nursing after fracture surgery was established for each patient. The members included one attending physician, one rehabilitation physician, and three nurses, among that the head nurse was the leader. Division of labor as follows: the attending physician was responsible for disease‐related professional knowledge explanation and prognosis progress assessment, the rehabilitation physician was responsible for functional exercise professional guidance, while three nurses respectively gave the advice of psychological nursing, diet nursing, prevention of complications nursing.
2) For patients who were randomly assigned to the Wechat group, the head nurse invited them to join the group and introduced the purpose, precautions, and basic rules of follow‐up of the Wechat group to each member and the patients.
3) The implementation of follow‐up and the specific contents of nursing include the following aspects.

### Answering Professional Questions about Diseases

Given the problems of postoperative pain, swelling, paresthesia, and wound healing, the attending doctor patiently answered them one by one. With the convenience of Wechat, patients could send relevant image data to doctors, which helps doctors more accurately grasp the location and general situation of symptoms, and give more targeted answers and guidance.

### Rehabilitation Training Guidance

Rehabilitation doctors make functional rehabilitation exercise plans according to each patient's particular needs. Through regular Wechat group reminders, patients could be changed from passive exercise to active exercise, from simple movements to complex movements, and from non‐weight‐bearing to weight‐bearing. Simultaneously, the intensity and time of exercise could be gradually and standardized increased.

### Psychological Care

One nursing staff was responsible for giving patients the necessary care and consideration after surgery, laying the foundation for establishing a good doctor–patient, nurse–patient relationship. At the same time, patients' negative emotions must be eliminated promptly and effectively. Strengthen the trust of patients and their families in the team of doctors and nurses through appropriate encouragement. Finally, the patient's subjective initiative is improved.

### Diet Nursing Care

Eating was typically encouraged. For patients with underlying conditions, such as diabetes, high blood pressure, etc., necessary special diets need to be maintained and supervised, such as diabetic, low salt and low‐fat, etc. In addition, high‐quality protein, crude fiber food and necessary calcium should be adequately and appropriately supplemented, which was conducive to average bone growth.

### Nursing for the Prevention of Complications

In Wechat group, one of the specialized nurse regularly sends attention points on the common postoperative complications of distal radius fractures and gives the related health education, such as median nerve irritability, infection of the incision, stiff joints, and traumatic arthritis, once appear associated complications, to respond promptly, and to give patients a regular and accurate treatment measure further. Once patients had related complications, regular, accurate further treatment measures should be given.

*Control Group*: The patients were followed up and guided by traditional methods, including regular telephone follow‐up and reminder and outpatient follow‐up. The patients were given rehabilitation guidance, question‐answering and psychological counseling.

*Specific Strategies*: The doctors, rehabilitators and nurses in charge of follow‐up and postoperative guidance were the same as those in the Wechat group. According to their respective functions and the specific situation of each patient, specific plans or notification forms such as rehabilitation exercise, dietary care, psychological counseling and complication prevention strategies were formulated before discharge. Patients were instructed to strictly follow them after discharge. If there are more questions, patients can seek answers through the phone or the clinic.

### Evaluation Index and Evaluation Method

*Regular Follow‐Up Rate*:From the patient's discharge after surgery, the patient was required to complete the review at the three time points of 1 month, 3 months, and 6 months after surgery to the surgeon's clinic. If the patient failed to return on time or could not be contacted due to various reasons during this period, the regular follow‐up was regarded as incomplete.

*Satisfaction*: A targeted satisfaction questionnaire was developed for the two groups of patients to evaluate the professional level, service attitude, health guidance, psychological counseling, and humanistic care of the medical staff, which was divided into three grades: satisfaction, average, and dissatisfaction. All questionnaires were collected and analyzed by staff not involved in this study.

*Postoperative Functional Rehabilitation Index*:Garland and Werley's scale was used to evaluate the postoperative function of patients,^[^ ^10^ ^]^ and the exercsie of self‐care agency score (ESCA)^[^ ^11^ ^]^ was used to assess the self‐care ability of patients in the two groups at 3 months after surgery. All scales were completed during the outpatient review. At the same time, the incidence and types of postoperative complications in the two groups were also analyzed.

### Statistical Analysis

SPSS 22.0 software was used for statistical analysis. Quantitative data were expressed as mean ± standard deviation (x ± s), and qualitative data were expressed as rate or percentage (%). The *χ*2 test was used for the qualitative data of the two groups. The “continuous correction” value was used when the expected value in any cell was <5 but the sample size was >40. The “Fisher exact two‐sided” value was used when the expected value in any cell was <1 or the sample size was <40. And the “Pearson Chi‐square two‐sided” value was used when there was no such situation. Mann‐Whitney U test was used for multiple groups of qualitative data, and three decimal places were reserved. The significance test level was *p* < 0.05 as the difference was statistically significant.

## Conflict of Interest

The authors declare no conflict of interest.

## Data Availability

The data that support the findings of this study are available from the corresponding author upon reasonable request.
